# Evaluating a Tailored Quality Improvement Intervention to Improve Vaccination Coverage in Sydney Residential Aged Care Facilities

**DOI:** 10.3390/vaccines14020171

**Published:** 2026-02-12

**Authors:** Courtney McGregor, Lauren Tillman, Lisa Maude, Karen Chee, Caitlin Swift, Leigh McIndoe, Mark Ferson, Brendan Goodger, Kira Wright, Vicky Sheppeard

**Affiliations:** 1 South Eastern Sydney Local Health District Public Health Unit, Sydney, NSW 2031, Australia; lauren.tillman@health.nsw.gov.au (L.T.); lisa.maude@health.nsw.gov.au (L.M.); karen.chee@health.nsw.gov.au (K.C.); caitlin.swift@health.nsw.gov.au (C.S.); leigh.mcindoe@health.nsw.gov.au (L.M.); mark.ferson@health.nsw.gov.au (M.F.); vicky.sheppeard@health.nsw.gov.au (V.S.); 2 School of Population Health, University of New South Wales, Sydney, NSW 2052, Australia; 3 Central and Eastern Sydney Primary Health Network, Sydney, NSW 2020, Australia; b.goodger@cesphn.com.au (B.G.); k.wright@cesphn.com.au (K.W.); 4 School of Public Health, University of Sydney, Sydney, NSW 2050, Australia

**Keywords:** vaccination in aged care, residential aged care facility, vaccination coverage reporting, vaccine-preventable diseases, quality improvement, quasi-experimental methods

## Abstract

Background/Objectives: Aged care residents are highly vulnerable to vaccine-preventable diseases. Despite recommendations and funding under Australian programs, vaccination rates among residents for COVID-19, influenza, pneumococcal and shingles remain sub-optimal. The aim of this work was to assess if tailored quality improvement interventions would improve vaccination coverage in aged care residents. Methods: This was a quality improvement initiative evaluated using a quasi-experimental pre–post design. Building on previously identified barriers and enablers, a package of interventions and resources was developed to support consent processes, vaccination planning, and tracking. Pre- and post-intervention vaccination coverage was assessed using resident lists from participating aged care facilities and data extracted from the Australian Immunisation Register (AIR) at two time points, 14 months apart. A process evaluation survey was distributed to RACF staff. Results: Of the 6964 residents listed, 5153 (74%) remained registered in AIR when data was extracted post-intervention. Shingles showed the greatest improvement in absolute difference (+23.4%), followed by pneumococcal (+14.2%) and influenza (+10.9%), despite a high baseline of 68.5%. COVID-19 coverage declined by 7.4% when applying a 6-month reporting interval. Twenty-five staff completed the process evaluation survey; 45% of respondents identified discrepancies between AIR data and internal records, indicating underreporting by external providers. Interventions including the consent template and vaccination tracker were reported as useful and were used to support local vaccination. Conclusions: This quality improvement initiative improved coverage for three of the four recommended and funded vaccines for RACF residents and demonstrated the value of tailored interventions informed by consumer and provider feedback. The approach potentially offers a scalable model for improving vaccination rates in aged care across Australia.

## 1. Introduction

Vaccination remains the most effective strategy for preventing hospitalisation and death among older adults [1], particularly those residing in residential aged care facilities (RACFs). Older adults in RACFs face heightened vulnerability to infectious disease due to advanced age, comorbidities [2,3], functional and cognitive decline [3], and the shared close-contact living environment [2,3]. Consequently, the burden of infectious disease is substantially higher among adults living in long-term residential care settings compared with those in the community [2,3]. With populations ageing globally, there is a pressing need to prioritise preventative measures that support healthy ageing, rather than relying on reactive approaches [4]. Vaccination plays a critical role in reducing infection risk, preventing complications, and lowering the likelihood of hospitalisation from vaccine-preventable disease [5,6].

Residents in RACFs are a priority population under the Australian Government’s National COVID-19 Vaccination Program [7] and National Immunisation Program (NIP) [8]. The four vaccines recommended and funded by these programs for older adults are COVID-19, influenza, pneumococcal and shingles. All vaccinations provided under these programs must be recorded in the Australian Immunisation Register (AIR), a centralised register which holds details of vaccines received by all Australian residents [9]. Regular assessment and reporting of population coverage is undertaken by local, state and national health agencies using the AIR data.

There are currently no national coverage targets for adult vaccinations in Australia, with this considered in the Implementation Plan of the National Immunisation Strategy 2025–2030 [10]. In the state of New South Wales (NSW), where South Eastern Sydney Local Health District (SESLHD) is located, the NSW Immunisation Strategy 2024–2028 has established targets for influenza and shingles vaccination for adults aged ≥65 years, and it dictates that uptake of all four recommended and funded vaccines should be monitored in RACFs [11].

Of the four vaccines, only COVID-19 vaccination coverage is routinely reported at a national level specifically for aged care residents. As of 8 October 2025, only 52.7% of aged care facility residents across Australia had received a COVID-19 dose in the preceding 6 months [12], as recommended [13]. Coverage data for the other vaccines in RACF settings remains limited [14]. One recent Australian study reported that 92.6% of RACF residents had received an influenza vaccine in the previous two years, 38.3% had received a pneumococcal vaccine, and only 16.8% had received a shingles vaccine [15]. These findings align with a study of RACFs in the state of Victoria, which reported coverage of 86.8% for influenza, 32.5% for pneumococcal vaccine, and 19.3% for shingles [16]. While these influenza coverage rates in RACF residents meet NSW Health targets, shingles coverage remains well below the target of 70% [11], and the current COVID-19 and pneumococcal coverage rates are considered suboptimal.

In response to gaps in vaccination coverage among residents of RACFs, the SESLHD Public Health Unit (PHU), in partnership with the Central and Eastern Sydney Primary Health Network (CESPHN), implemented a quality improvement initiative in 2024 aimed at improving vaccination coverage among RACF residents.

The first part of our initiative involved gathering information on the enablers and barriers to vaccination of RACF residents, from the perspectives of vaccination providers, RACF staff, residents and their family members. Through this work, we identified operational, communication, coordination and financial enablers and barriers, which have been previously described [14]. We used these findings to develop a package of targeted interventions and resources to support vaccination uptake in this setting. We then sought to determine if implementation of this tailored, multicomponent quality improvement intervention would improve age-recommended vaccination coverage among residents for COVID-19, influenza, pneumococcal and shingles.

This paper describes both the package of interventions and supportive resources that we developed and their impacts as assessed by pre- and post-intervention resident vaccination coverage data and an evaluation survey of RACF staff.

## 2. Materials and Methods

### 2.1. Context

SESLHD is located in metropolitan Sydney and comprises 97 RACFs accommodating nearly 8000 residents. The district serves an overall population of almost one million people, approximately 30% of whom are from non-English-speaking backgrounds [17].

### 2.2. Study Design

This quality improvement initiative was evaluated using a quasi-experimental pre–post design [18]. This approach was selected to evaluate changes in routine clinical practice and to describe system-level shifts in vaccination coverage under real-world conditions. Based on previously identified barriers and enablers collected through stakeholder questionnaires between June and September 2024 [14], a package of interventions and supportive resources was developed and implemented across participating RACFs between August and September 2024. Educational sessions and forums for RACF staff were run between August 2024 and July 2025. The package included tools to support consent processes, vaccination planning, and tracking. These are described below.

This study is reported in accordance with the Standards for Quality Improvement Reporting Excellence (SQUIRE 2.0) guidelines [19].

### 2.3. Participants

All 97 RACFs located within SESLHD were invited by the PHU to participate in the initiative. Further details of RACF recruitment have been previously described [14]. Resident-level characteristics available for analysis were limited to age, sex, and Indigenous status, as captured in facility line lists and the AIR.

#### 2.3.1. Inclusion Criteria

Participants were included in the analysis if they were permanent residents listed on the line lists submitted by participating RACFs and were eligible by age to receive government-funded vaccinations (Table 1).

#### 2.3.2. Exclusion Criteria

Resident records were excluded if they appeared in the AIR unmatched output post-intervention (and were therefore presumed to have died during the intervention period), or if the resident had been admitted to an RACF after the initial line list was submitted.

### 2.4. Package of Interventions

#### 2.4.1. Online Consent Template

An online consent form template was developed using Microsoft Forms to support RACFs in organising vaccination clinics (Consent template S1). The consent form included all four vaccines listed in Table 1 and was designed to be localised by facilities and distributed electronically to residents or their decision-makers for completion. The form included hyperlinks to the low-literacy factsheets described below. Upon submission, responses populated a Microsoft Forms database accessible to the facility, recording resident details and vaccination intentions.

The form was designed to be completed once per resident stay at the RACF and captured specific consent for each vaccine. This included consent for the single dose or course recommended for pneumococcal and shingles vaccines. It also covered 6-monthly COVID-19 and annual influenza vaccines, which require repeated administration during the resident’s stay. Consent could be revoked at any time by notifying the facility. Vaccination providers retained responsibility for assessing precautions, contraindications and confirming consent at the time of vaccination, in line with usual clinical practice.

#### 2.4.2. Vaccination Action Plan Toolkit

Findings from the RACF staff questionnaires informed the development of a vaccination action plan toolkit. Topics included guidance on accessing residents’ immunisation histories via the AIR and My Health Record (MHR), using the vaccination tracker register (described below), communicating with residents and families about vaccination, obtaining consent, coordinating vaccination providers and clinics, maintaining cold chain requirements for vaccine storage, and managing adverse events following immunisation (AEFI). Each participating RACF received an action plan toolkit containing general information on all of these topics, along with a summary of the site-specific barriers identified during each RACF’s initial questionnaire and recommendations tailored to address those barriers.

#### 2.4.3. Vaccination Tracker Register

The vaccination tracker register (‘tracker’) was developed as a practical tool to track when individual residents were due for vaccinations and to calculate facility-level coverage for each of the four vaccines. The tracker was designed as a Microsoft Excel spreadsheet with inbuilt formulas to perform these calculations (Tracker template S2).

Participating RACFs provided a line list of the name, date of birth and Medicare number (unique national universal health care identifier) of their permanent residents. We retrieved immunisation records for each resident from the AIR using the AIR012A database exchange report, which enables vaccination providers to check AIR records for a list of individuals [20]. The AIR012A report generates outputs including individual details (IH), immunisation history (VC), and unmatched (UM) data, which identifies errors in information entered into the AIR. Unmatched data were checked by verifying resident details with RACF staff, and the report was re-run using corrected information to ensure all permanent residents were included.

We created each facility tracker by applying a Microsoft Excel Visual Basic for Applications (VBA) macro to the consolidated IH and VC outputs from the AIR012A report. The inbuilt formulas used the last vaccination date for each vaccine antigen and eligibility criteria shown in Table 1 to calculate the vaccination status (up to date or overdue) of residents for each vaccine. Eligibility for vaccination was calculated based on age and Indigenous status as it was recorded in the AIR.

The trackers were returned to each RACF, populated with each resident’s vaccination status for the four vaccines, which enabled staff to identify overdue residents and organise vaccinations. When a vaccine was administered, staff could manually enter the new vaccination date into the tracker, which automatically recalculated individual vaccination status and facility-level coverage. RACF staff could then use the tracker on an ongoing basis to monitor vaccinations at both individual and facility levels. RACFs could also manually add residents along with their vaccination history, or remove residents as required.

### 2.5. Supportive Resources

#### 2.5.1. Low Literacy, Translated Factsheets

Factsheets were created by the PHU for each of the four vaccines using low-literacy level language [21] and are publicly available. Factsheets contained information on the disease, how the vaccine protects older adults, recommended timing, common side effects, and where to find further advice. Each factsheet was also translated into eight priority languages [22] that had been identified by RACF staff as the most common languages other than English used by their residents.

#### 2.5.2. Vaccination Policy Template

A vaccination policy template was developed to assist RACFs in creating their own policies, particularly for facilities without an existing framework. The template provided high-level guidance that could be tailored to each individual facility. It outlined principles, rules, and expectations for staff to ensure consistency and accountability. It included guidance on staff roles and responsibilities, vaccination recommendations for older adults, processes for checking residents’ immunisation history and monitoring when vaccinations were due, consent procedures, identification of vaccination providers, cold chain requirements for vaccine storage and management of breaches, organisation of vaccination clinics, and protocols for managing anaphylaxis and adverse events following immunisation (AEFI).

#### 2.5.3. Instructional Videos

To support RACF staff to navigate the interventions, three instructional videos were created using Microsoft Clipchamp software explaining how to use the online consent template, vaccination action plan toolkit, and vaccination tracker register.

These videos were distributed to participating RACFs and are hosted on the PHU website [22] for RACF staff to access at any time.

#### 2.5.4. Education Sessions and Forums for RACF Staff

We delivered a series of online and face-to-face education sessions for RACF staff between August 2024 and July 2025.

Two one-hour education sessions were delivered online in August and September 2024. Topics included vaccination recommendations, accessing immunisation history via AIR and MHR [23], communicating with vaccine-hesitant individuals using Sharing Knowledge About Immunisation (SKAI) resources [24], cold chain management [25], and AEFI reporting [26].

These education sessions were followed by two one-hour online forums in October 2024. The forums introduced the developed interventions and resources to address the reported vaccination barriers.

Between January and June 2025, on-site cold chain audits were conducted with RACFs that held NSW state vaccine accounts to ensure staff were following the National Vaccine Storage guidelines: Strive for 5 [25].

A one-hour online roundtable forum was later held in July 2025 to further discuss the package of interventions and resources and determine if RACF staff required additional support. Findings and recommendations are documented in a separate report, which has been disseminated to state and federal government bodies responsible for health and aged care policies [27].

The interventions and resources were refined over the course of the 14-month period in response to ongoing consultation and feedback from RACF staff in the evaluation survey, meetings and education sessions.

### 2.6. Assessment of Vaccination Coverage 

Vaccination coverage was assessed across the combined cohort of residents from SESLHD RACFs using demographic characteristics available in the AIR (age and Indigenous status).

Vaccination histories were extracted from the AIR between 30 September and 3 October 2025 and used to assess coverage at two time points: 1 August 2024 (pre-intervention) and 3 October 2025 (post-intervention).

Vaccination coverage for each antigen was calculated descriptively, with results reported as absolute and relative differences between the two time points. Facility-level coverage was calculated for each RACF and summarised using medians and interquartile ranges to describe inter-facility variability. As the dataset represented the near-complete population of SESLHD RACF residents, analyses describe observed system-level changes rather than sample-based estimates. Consistent with SQUIRE 2.0 guidance for non-randomised quality improvement studies, inferential statistical testing was not undertaken.

Residents were considered vaccinated if they met the age eligibility (refer to Table 1) and the following criteria:

- COVID-19: receipt of a vaccine within the previous 6 months; a 7-month interval was also examined to account for potential operational delays.
- Influenza: receipt of a vaccine within the previous 12 months
- Pneumococcal: receipt of one dose of a pneumococcal conjugate vaccine (Prevenar 13)
- Shingles: receipt of two doses of Shingrix.

Residents who had previously received Zostavax were considered unvaccinated.

All data cleaning, manipulation and analysis were conducted in R Statistical Software (version 4.5.1 Patched) [28].

### 2.7. Assessment of the RACF Staff Process Evaluation Survey

A process evaluation survey was created in REDCap to gather feedback from RACF staff on the useability and usefulness of the package of interventions and supportive resources developed by the PHU, as well as overall satisfaction with the quality improvement initiative (Survey S3). All staff involved in the initiative, from the 90 participating RACFs, were invited to take part. Invited participants included a range of clinical and corporate managers. The survey link was shared via email multiple times between 5 December 2024 and 3 February 2025. Survey responses were exported to Microsoft Excel, and quantitative data from closed-ended questions are presented as percentages.

### 2.8. Ethical Considerations

Consent for the RACF staff process evaluation survey was embedded within the REDCap questionnaire, with completion taken as consent. Individual resident consent was not obtained for vaccination coverage assessment, as this work was conducted as a quality improvement initiative and involved routine Public Health Unit use of the AIR data and RACF resident line lists for public health purposes. The work was approved as a quality improvement initiative by the South Eastern Sydney Local Health District Human Research Ethics Committee (QAQI/11March2025/R2).

## 3. Results

### 3.1. Vaccination Coverage Outcomes

Ninety RACFs accepted the invitation to participate in this quality improvement initiative, while seven declined. Of the 90 participating RACFs, 85 (94%) provided a resident line list between July and September 2024. These line lists included 6964 permanent residents. When vaccination data were re-extracted from the AIR on 3 October 2025, 5153 (74.0%) remained listed in the AIR and were included in the analysis. Residents who did not appear in the AIR at follow-up (*n* = 1811) were considered deceased and were excluded from both the pre- and post-intervention analyses.

The demographic characteristics of the residents are shown in Table 2.

#### 3.1.1. Pre- and Post-Intervention Vaccination Coverage

Pre- and post-intervention vaccination coverage for the included 5153 residents is presented in Table 3. Coverage for shingles vaccination showed the largest improvement, increasing by 23.4%, followed by pneumococcal vaccine coverage (14.2%). Influenza vaccine coverage increased by 10.9%, despite an already high baseline of 68.5%. COVID-19 vaccination coverage declined by 7.4% to 53.9% when using a 6-month interval. When a 7-month interval was applied, COVID-19 coverage was higher at 66.0% pre-intervention and 64.8% post-intervention.

The largest relative increases were observed for shingles (+585%) and pneumococcal (+64%), followed by influenza (+15.9%), reflecting improvements from a low baseline for shingles and pneumococcal vaccines.

#### 3.1.2. Facility-Level Variation in Vaccination Coverage

Facility-level vaccination coverage varied across RACFs at both time points (Table 4). Median coverage increased for influenza, pneumococcal, and shingles vaccines and decreased for COVID-19. Interquartile ranges show variability between facilities, with the widest variation at follow-up for shingles vaccination.

### 3.2. Findings from the RACF Staff Process Evaluation Survey

A total of 25 RACF staff responded to the staff process evaluation survey. All reported they were very satisfied (21/25, 84%) or somewhat satisfied (4/25, 16%) with the overall quality improvement initiative.

The vaccination tracker register was reported to be useful by 23 (92%) respondents. As a direct result of receiving the vaccination tracker register, 23 (92%) respondents arranged a vaccination clinic at their facility. After comparing the AIR data with their internal vaccination records, 11 (45%) respondents noted that their external vaccination providers had not entered resident vaccination encounters into the AIR.

Sixteen (64%) respondents found the online consent template useful, and 14 (56%) reported they intended to use it. The vaccination action plan toolkit and the instructional videos to support resource use were each reported as helpful by 22 (88%) respondents.

## 4. Discussion

This quality improvement initiative suggests that targeted, facility-focused interventions and resources were associated with improved vaccination coverage for residents in RACFs. The findings underscore the need for RACF-specific tools to support consent, vaccination planning, and tracking of vaccine uptake, alongside supporting RACF staff through training and education.

Our previous work identified that organising consent was a major barrier to resident vaccination in RACFs [14]. The online consent template was developed to improve accessibility and streamline timely consent processes. Although frontline RACF staff recognised the potential benefits of it, uncertainty regarding its legal validity and implementation processes may have limited adoption [27]. To support broader use, endorsement at the state or national government level may be more effective than promotion solely at the local health district level. A relevant precedent is the implementation of the online Consent and Records Management for Immunisation (CaRMI) system adopted in the NSW school vaccination program from 2023. CaRMI was introduced by the NSW Government to improve the management of consent records, streamline clinic scheduling, and facilitate timely communication with families [29].

Building on the need for system-level support for consent processes, our vaccination action plan toolkit was uniquely tailored to the RACF setting, offering site-specific recommendations and practical support for implementation of vaccination programs. Unlike existing toolkits [30,31], ours focused specifically on addressing identified operational and coordination barriers in RACFs. This specificity enhances its relevance and usability for aged care staff, addressing the unique challenges faced in RACFs that may not be fully captured in general public health toolkits.

Previous studies have highlighted the urgent need for effective vaccination tracking systems in aged care settings, noting that well-designed digital tools can rapidly adapt to changing eligibility criteria and support more timely vaccination [32]. In the absence of such systems, we developed the vaccination tracker register. The tracker supported local planning, as evidenced by many facilities subsequently conducting vaccination clinics. However, because the tracker was not linked to the AIR, it could not account for reporting delays or vaccinations administered after its initial distribution, and it required manual updating by RACF staff. Limitations also exist within the AIR itself: reports are typically linked to individual providers or medical practices rather than RACFs [20], are not presented in a user-friendly format, and required substantial manipulation in this initiative (including a Microsoft Excel VBA macro) to generate usable information.

Prior evidence indicates that digital reminders are more effective than non-digital approaches in improving vaccination coverage; however, RACF electronic care monitoring systems lack functionality to track multiple vaccines, often relying on separate reports, multiple systems or manual calculations [14]. Together, these challenges underscore the need for an integrated age-care-specific digital system that links directly to centralised databases [32] like the AIR and incorporates automated reminders [33]. This would better support facility-wide monitoring and aligns with national priorities to improve the use of immunisation strategies and performance monitoring [10].

Low health literacy is well known to significantly affect an individual’s ability to access, understand, and use health information [34]. Despite changes over time, challenges remain. In 2006, only 41% of Australians aged 15–74 were assessed as having adequate or higher health literacy skills, with the remainder classified as having less than adequate or very low literacy [35]. In 2018, 12% reported difficulty finding reliable health information and 8% had trouble understanding it [34]. More recently, 44% of Australians read at a literacy level of 1–2 out of 5, which is considered low [21]. These figures, though derived from different methodologies, consistently highlight a substantial gap in health communication. Given the district’s multicultural and socioeconomically diverse population [36], accessible and inclusive communication is essential. Barriers such as fear of side effects, language, literacy, and poor communication hinder vaccine uptake among RACF residents and migrant groups, particularly those who speak languages other than English [14,37], while tailored education, translated resources, and supportive vaccination policies are recognised enablers for these populations [14,37]. Our initiative addressed these factors by developing low-literacy, translated factsheets, providing accessible health information to support consent processes.

It is important to recognise that effective implementation of new innovations in aged care settings relies on enablers such as appropriate staff training, knowledge sharing, and facilitation [38]. To support adoption of the package of interventions, a series of education sessions—delivered both online and in person—was implemented and likely contributed to improved uptake, as evidenced by the reported usefulness of, and intention to use, intervention components reported in the staff process evaluation survey. This approach aligns with recent evidence demonstrating that in-person education significantly improves RACF staff knowledge, skills, and confidence [39]. Together, these findings underscore the importance of practical resources and on-site education in supporting successful implementation and optimising outcomes for residents.

In this resident cohort, shingles vaccination demonstrated the largest improvement in coverage, followed by pneumococcal. Previous work has shown that shingles and pneumococcal vaccinations were not routinely tracked by RACF staff [14], which may explain the greater improvements observed for these vaccines as staff became aware of coverage gaps. Reported reasons for lower overall uptake of these two vaccines among older adults include perceptions that vaccination was unnecessary, lack of recommendation from a doctor, limited awareness, and uncertainty about benefits [40]. The very low pre-intervention coverage for shingles may partly reflect the recent change to the Australian shingles vaccination schedule, with Zostavax replaced by Shingrix in November 2023 [41]. There may also be an assumption that residents were vaccinated prior to admission given that the NIP eligibility age is younger than the age of most RACFs residents [16]. These factors highlight the importance of effective vaccination tracking systems and targeted campaigns for vaccines with lower uptake, including clear communication of schedule changes, consistent with recommendations from previous authors in the Australian setting [15].

Recently reported behavioural and social drivers of influenza vaccination intention included personal responsibility for health, reminders, influence of the surrounding community and personal decision-making [42]. Adults who identified themselves as the primary decision-maker were 3.1 times more likely to want a flu vaccine compared to those who identified someone else as the decision-maker [42]. This is an important consideration in the aged care setting, where not all residents make their own vaccination decisions, which may contribute to lower vaccination rates across all vaccines, consistent with findings from our previous work [14].

Despite gains in vaccination coverage for pneumococcal, shingles and influenza, COVID-19 coverage calculated at a 6-month interval declined, highlighting the ongoing challenges facilities face in maintaining up-to-date COVID-19 vaccination. Factors such as vaccine fatigue [39], fear of side effects [14,43], changing health advice and misinformation [39,43] may have contributed to this decline. However, during the same period (August 2024 to October 2025), national COVID-19 vaccination coverage in RACFs remained relatively stable at 51.3% and 52.7%, respectively [12]. While our resident cohort did not achieve gains in COVID-19 coverage, pre-intervention rates were 10% higher than reported national rates. Furthermore, when using a 7-month reporting interval to allow for a potential lag before administering the next dose, coverage was higher by 4.7% and 10.9% pre- and post-intervention, respectively. This suggests that applying a one-month grace period for national reporting may provide a more accurate and potentially improved picture of COVID-19 vaccination coverage among aged care residents across Australia, and the larger decline in our COVID-19 coverage at a 6-month interval may simply be due to scheduling intervals.

Provider endorsement [14,39,43] and clearer, more consistent communication around the six-month dosing interval are essential to support timely vaccine delivery and minimise preventable declines in coverage [6]. Further work is needed to develop practical frameworks to overcome known barriers to COVID-19 vaccine acceptance, particularly in aged care settings given the vulnerability of this population.

In addition to coverage challenges, reporting accuracy emerged as an issue with underreporting of resident vaccinations to the AIR by external vaccination providers. Underreporting to the AIR has been previously documented, with discrepancies in childhood coverage ranging from 5% to 14% [44] and up to 44% for adult vaccinations [45]. Despite mandatory reporting requirements under the Australian Immunisation Register Act 2015 [9,46], it was reported that some vaccination providers failed to upload immunisation encounters.

These issues are likely not unique to the facilities involved in this initiative and may reflect a systemic challenge across the aged care sector. Such underreporting may partly explain the low COVID-19 and influenza vaccination coverage rates for RACFs regularly reported by the Australian Government Department of Disability, Health and Aged Care [47,48]. Previous authors have recommended a multi-faceted approach to improving data accuracy, including provider education, structural changes to the AIR platform, and reducing barriers to access the AIR [49]. The lack of direct access to the AIR by RACFs prior to, and for most of, the intervention period meant that RACF staff had to rely on external vaccination providers to report vaccinations to the AIR. From May 2025, all RACFs were granted direct access to the AIR following our advocacy [14], enabling RACFs to enter or audit vaccination data and improving the accuracy and timeliness of coverage reporting.

### 4.1. Strengths and Limitations

A key strength of this quality improvement initiative was the iterative refinement of resources in response to RACF staff needs, which improved their relevance and usability. Another strength was the ability to calculate accurate, vaccine-specific coverage across four vaccines using a verified list of current RACF residents. With a substantial cohort size (*n* = 5153) of the almost 8000 residents in the district, these estimates are likely more reliable than Commonwealth-reported coverage, which relies on snapshots of residents in funded positions [47,48]. Importantly, pneumococcal and shingles vaccination coverage is not routinely reported for RACF residents, and, to our knowledge, this is the first NSW study to publish coverage data for these vaccines in a large RACF resident cohort.

This work also has several limitations. As is common in real world quality improvement initiatives, the package of interventions and resources were provided as a bundle, and delivery of individual intervention components was not monitored at the RACF level. This limits causal attribution and hinders interpretation of the substantial inter-facility variation observed. While the package of interventions and resources was associated with meaningful coverage improvement for three of the four vaccines, wide variation between RACFs persisted, suggesting differential uptake and/or effectiveness across facility contexts.

Coverage estimates relied on resident line lists initially provided by RACFs, resulting in the exclusion of 1811 residents who died during the intervention period and were therefore not included in pre- and post-intervention coverage estimates. This may have introduced attrition bias if deceased residents were less likely to be vaccinated. Additionally, demographic characteristics beyond age, sex, and Indigenous status were not collected, limiting assessment of person-level determinants of uptake. Reliance on AIR data rather than internal RACF records-used in a previous Australian study [15] introduces further uncertainty, as underreporting to the AIR is a known issue and was observed during this initiative. Consequently, baseline coverage may have been underestimated and apparent improvements overstated. Potential confounders, such as concurrent vaccination campaigns, may also have influenced coverage outcomes; however, to the authors’ knowledge, no additional campaigns occurred during the intervention period beyond routine NSW Health and Australian Government activities. Finally, the low number of respondents to the RACF staff process evaluation survey (*n* = 25/90) introduces the possibility of non-response bias, which may limit the generalisability of the evaluation findings.

### 4.2. Future Work

Future work should focus on understanding how individual RACFs operationalise the package of interventions and resources, and on identifying contextual, organisational, and workforce factors influencing uptake, particularly given the persistent inter-facility variation observed. Facilities with lower coverage may require more tailored or intensive implementation support to achieve comparable gains.

Embedding and adapting the intervention package to accommodate diverse operational contexts and cultural needs—including facilities outside SESLHD—will be essential. Targeted approaches that address the specific barriers faced by lower-performing facilities may help reduce variability and promote more equitable vaccination coverage across the sector. Collaboration with corporate aged care organisations will also be critical to ensure alignment with organisational priorities and operational constraints. Further research should examine the scalability, sustainability, and relative impact of individual intervention components across facilities with different levels of resources, workforce capacity, and resident demographics to optimise their impact for this highly vulnerable population.

## 5. Conclusions

Over a 14-month period, this quality improvement initiative demonstrated improved vaccination coverage for three of the four recommended and funded vaccines for residents of participating RACFs. Engaging with RACF staff and residents is key to identifying vaccination barriers and enablers and informs tailored interventions. The tools and strategies that were developed empowered RACF staff to monitor and arrange vaccinations for residents in a sustainable manner. The suite of interventions could be adopted by other RACFs, potentially offering a scalable model of care to improve vaccination rates for RACF residents across Australia.

## Figures and Tables

**Table 1:** Vaccination recommendations for older adults in Australia for vaccines funded through the Australian National COVID-19 Vaccine Program [7] and the National Immunisation Program (NIP) [8,13].

Vaccine	Recommended Timing of COVID-19 Vaccines Booster Doses for Older Adults		
COVID-19	Every 6 months for all adults ≥ 75 years old Every 6 months for RACF residents ≥ 65–74 years old Every 12 months for general population ≥ 65–74 years old		
**Vaccine**	**Recommendation**	**Age Vaccine Is Funded From**	
		Non-Indigenous	Indigenous
Influenza	Annual single dose	65 years	6 months
Pneumococcal	Single dose of Prevenar 13 (13vPCV) for all adults PLUS 2 doses of Pneumovax 23 (23vPPV) for all Indigenous adults and for non-Indigenous adults with vulnerable conditions	70 years	50 years
Shingles	2 doses Shingrix	65 years	50 years

**Table 2:** Demographic characteristics of the RACF resident cohort as recorded in the AIR.

Characteristic	Resident Cohort (*n* = 5153)
Age, years (as of 3 October 2025)	
Median (range)	88 (44–109)
Mean (SD)	86.7 (±8)
Gender, *n* (%)	
Male	1727 (33.51%)
Female	3426 (66.49%)
Indigenous status, *n* (%)	
Indigenous	31 (0.60%)
Non-Indigenous	5118 (99.32%)
Missing/Unknown	4 (0.08%)

**Table 3:** Pre- and post-intervention vaccination coverage for SESLHD RACF residents.

Vaccine	Pre-Intervention *N*	Pre-Intervention (%)	Post-Intervention *N*	Post-Intervention (%)	Absolute Difference (%)	Relative Change (%)
COVID	3159	61.3	2775	53.9	−7.4	−12.1
Influenza	3528	68.5	4090	79.4	+10.9	+15.9
Pneumococcal	1109	22.2	1832	36.4	+14.2	+64.0
Shingles	203	4.0	1403	27.4	+23.4	+585.0

**Table 4:** Facility-level pre- and post-intervention vaccination coverage for SESLHD.

Vaccine	Median Pre-Intervention (%)	Median Post-Intervention (%)	IQR Pre (Q1–Q3, %)	IQR Post (Q1–Q3, %)
COVID	70.2	63.5	48.5–77.5	29.4–74.3
Influenza	75.1	81.2	55.2–86.4	75.2–88.1
Pneumococcal	15.5	26.8	11.8–23.3	15.9–54.5
Shingles	2.3	19	0.0–5.0	5.0–43.9

IQR = interquartile range (Q1–Q3) across facilities. Median and IQR represent facility-level vaccination coverage percentages.

## Data Availability

The data presented in this study are available upon request from the corresponding author. Due to ethical restrictions, they are not publicly available.

## Supplementary Materials

The following supporting information can be downloaded at: https://www.mdpi.com/article/10.3390/vaccines14020171/s1, Consent template S1: online consent template. Tracker S2: vaccination tracker register template. Survey S3: RACF staff evaluation survey.

## Abbreviations

The following abbreviations are used in this manuscript:

NSW	New South Wales
CESPHN	Central and Eastern Sydney Primary Health Network
SESLHD	South Eastern Sydney Local Health District
PHU	Public Health Unit
AEFI	Adverse Event Following Immunisation
AIR	Australian Immunisation Register
VBA	Visual Basic for Applications
SKAI	Sharing Knowledge About Immunisation
MHR	My Health Record
CaRMI	Consent and Records Management for Immunisation
