# Supplementary material for: Evaluating a Tailored Quality Improvement Intervention to Improve Vaccination Coverage in Sydney Residential Aged Care Facilities
Source: Vaccines (Basel). 2026 Feb 12;14(2):171. doi: 10.3390/vaccines14020171 (PMC12944912; doi:10.3390/vaccines14020171)
Supplement: Supplementary file 1 [file vaccines-14-00171-s001.zip › vaccines-4080568-supplementary -update/Consent template S1 online consent template.pdf]

Vaccination consent form for <Name of aged care facility> residents

Dear Residents and Families,

NSW Health recommends residents of aged care facilities receive up to four types of vaccine depending on your/their age - the COVID-19, influenza, pneumococcal and shingles vaccines.

These vaccines are free and protect older adults against common infections that can have serious health effects. Further information is provided in the links below.

To ensure these vaccines can be given on time, please complete this vaccination consent form as soon as you can. We will automatically receive the form when it is submitted.

On the scheduled day of vaccination each resident for whom we have consent will be assessed by the vaccination provider to check your/their suitability for vaccination. This includes overall health and any medical contraindications to vaccination (conditions or risk factors that make receiving a specific vaccine unsuitable). Medical contraindications to vaccination are rare.

If you have any questions or wish to withdraw consent at any time, please speak with a staff member.

Thank you for helping to protect the health and wellbeing of all residents in our facility.

1. Resident's name *

2. Resident's date of birth *

3. Are you completing this form on behalf of a resident? *

- ◯ Yes
- ◯ No

4. Your name (if completing the consent on behalf of a resident) *

5. Your contact number (if completing the consent on behalf of a resident) *

6. Your email (if completing the consent on behalf of a resident) *

7. I consent to my relative/the resident receiving the COVID-19 vaccine every 6 months.

   Please refer to information on COVID-19 infection and the vaccine here:
   https://www.seslhd.health.nsw.gov.au/sites/default/files/groups/Public_Health_Unit/RACF/COVID-19%20infection%20and%20vaccination.pdf
   *

   ◯ Yes

   ◯ No

8. I consent to my relative/the resident receiving the influenza vaccine every year.

   Please refer to information on influenza infection and the vaccine here:
   https://www.seslhd.health.nsw.gov.au/sites/default/files/groups/Public_Health_Unit/RACF/Influenza%20infection%20and%20vaccination.pdf *

   ◯ Yes

   ◯ No

9. I consent to my relative/the resident receiving the pneumococcal (Prevenar or Vaxneuvance) vaccine (if not already received).

   Please refer to information on pneumococcal infection and the vaccines here:
   https://www.seslhd.health.nsw.gov.au/sites/default/files/groups/Public_Health_Unit/RACF/Pneumococcal%20infection%20and%20vaccination.pdf *

   ◯ Yes

   ◯ No

10. I consent to my relative/the resident receiving the two pneumococcal (Pneumovax 23) vaccines (if eligible and not already received).

    Please refer to information on pneumococcal infection and the vaccines here:
    https://www.seslhd.health.nsw.gov.au/sites/default/files/groups/Public_Health_Unit/RACF/Pneumococcal%20infection%20and%20vaccination.pdf *

    ◯ Yes

    ◯ No

11. I consent to my relative/the resident receiving the two shingles (Shingrix) vaccines (if not already received).

    Please refer to information on shingles and the vaccine here:
    https://www.seslhd.health.nsw.gov.au/sites/default/files/groups/Public_Health_Unit/RACF/Shingles%20infection%20and%20vaccination.pdf *

    ◯ Yes

    ◯ No

12. I consent to receiving the COVID-19 vaccine every 6 months.

    Please refer to information on COVID-19 infection and the vaccine here:
    https://www.seslhd.health.nsw.gov.au/sites/default/files/groups/Public_Health_Unit/RACF/COVID-19%20infection%20and%20vaccination.pdf
    *

    ◯ Yes

    ◯ No

13. I consent to receiving the influenza vaccine every year.

    Please refer to information on influenza infection and the vaccine here:
    https://www.seslhd.health.nsw.gov.au/sites/default/files/groups/Public_Health_Unit/RACF/Influenza%20infection%20and%20vaccination.pdf *

    ◯ Yes

    ◯ No

14. I consent to receiving the pneumococcal (Prevenar or Vaxneuvance) vaccine (if not already received).

    Please refer to information on pneumococcal infection and the vaccines here:
    https://www.seslhd.health.nsw.gov.au/sites/default/files/groups/Public_Health_Unit/RACF/Pneumococcal%20infection%20and%20vaccination.pdf *

    ◯ Yes

    ◯ No

15. I consent to receiving the two pneumococcal (Pneumovax 23) vaccines (if eligible and not already received).

    Please refer to information on pneumococcal infection and the vaccines here:
    https://www.seslhd.health.nsw.gov.au/sites/default/files/groups/Public_Health_Unit/RACF/Pneumococcal%20infection%20and%20vaccination.pdf *

    ◯ Yes

    ◯ No

16. I consent to receiving the two shingles (Shingrix) vaccines (if not already received).

    Please refer to information on shingles and the vaccine here:
    https://www.seslhd.health.nsw.gov.au/sites/default/files/groups/Public_Health_Unit/RACF/Shingles%20infection%20and%20vaccination.pdf *

    ◯ Yes

    ◯ No

17. Your signature *

18. Date of signature *

This content is neither created nor endorsed by Microsoft. The data you submit will be sent to the form owner.

Microsoft Forms
