# Supplementary material for: Evaluating a Tailored Quality Improvement Intervention to Improve Vaccination Coverage in Sydney Residential Aged Care Facilities
Source: Vaccines (Basel). 2026 Feb 12;14(2):171. doi: 10.3390/vaccines14020171 (PMC12944912; doi:10.3390/vaccines14020171)
Supplement: Supplementary file 1 [file vaccines-14-00171-s001.zip › vaccines-4080568-supplementary -update/Survey S3 RACF staff evaluation survey.pdf]

Improving vaccination coverage in RACF residents - Evaluation survey

The Public Health Unit is hoping to find out how staff in aged care facilities found the vaccination assessment process by the Public Health Unit (PHU), and how useful the solutions developed by the PHU are.

Your answers are confidential, and will not be shared with other staff, residents or families at your facility.

You do not have to answer any questions if you do not wish to.

Thank you for participating in the project and taking the time to review this survey.

It should take up to 10 minutes to complete.

Your responses will help the PHU improve the vaccination rates in aged care facility residents.

What is the name of the RACF you work at? (optional) ______________________

I consent to participating in this survey.
- ◯ Yes
- ◯ No

What is your name or initials? (optional) ______________________

What is your role at the RACF?
- ◯ Facility manager
- ◯ Care manager
- ◯ IPC lead/coordinator
- ◯ Corporate level staff
- ◯ Registered nurse
- ◯ Other

What is your role? ______________________

Date completing the survey ______________________

This section asks about your initial assessment interview (or survey) by the Public Health Unit.

How was your initial assessment interview (or survey) with the Public Health Unit conducted?
- ◯ Face to face
- ◯ Video conferencing (using Microsoft Teams)
- ◯ Over the phone
- ◯ Completing online survey

Was this way of assessment satisfactory?
- ◯ Yes
- ◯ No

Please explain why not ______________________

Did you email the family survey link to your family distribution list?
- ◯ Yes
- ◯ No
- ◯ Don't know

What was the reason you did not email the family survey link?
For example, too busy, forgot, did not feel like the families would want to participate, other. ______________________

Were the topics discussed during the assessment useful?

	Useful	Neutral	Not useful
Staff awareness of age-recommended vaccines	◯	◯	◯
Access to Australian Immunisation Register (AIR)	◯	◯	◯
Tracking when residents are due for vaccinations	◯	◯	◯
Vaccine consent issues	◯	◯	◯
Accessing vaccination providers (e.g. GPs, pharmacists)	◯	◯	◯
Cold chain requirements	◯	◯	◯

If you found any of the topics "not useful", please explain why? ______________________

What other topics would you have liked to discuss? ______________________

This section asks about the solutions developed by the Public Health Unit to overcome vaccination barriers.

Was it useful for the Public Health Unit to provide a spreadsheet of the dates and vaccines received by each of your residents (COVID-19, influenza, pneumococcal and shingles vaccines) according to the information in the Australian Immunisation Register (AIR)?
◯ Yes
◯ No
◯ Don't know

How was it useful? ______________________

Why was it not useful? ______________________

Is the vaccination tracker developed by the Public Health Unit useful to track when your residents are due for their vaccinations?
◯ Yes
◯ No
◯ Don't know

How is it useful? ______________________

Why is it not useful? ______________________

Is your facility currently using or intending to use the vaccination tracker?	◯ Currently using<br>◯ Intending to use<br>◯ Not using<br>◯ Not intending to use<br>◯ Don't know
Will your facility be able to update the vaccination tracker when new residents are admitted or when residents receive vaccinations?	◯ Yes<br>◯ No<br>◯ Don't know<br>◯ We need more support
Please explain why you won't be able to use the vaccination tracker for new residents.	__________________________________
Did the information provided to you in the vaccination tracker spreadsheet prompt you to arrange a vaccination clinic for either the COVID-19, influenza, shingles or pneumococcal vaccines?	◯ Yes<br>◯ No<br>◯ Don't know
After being provided with the vaccination tracker, did you find that your usual vaccination provider (for example, GP or pharmacist) had not recorded resident vaccinations in the Australian Immunisation Register (AIR)?	◯ Yes<br>◯ No<br>◯ Don't know
Did you tell the PHU about the missing data in AIR?	◯ Yes<br>◯ No<br>◯ Don't know
Has this issue been addressed and resolved?	◯ Yes<br>◯ No<br>◯ Don't know
If you would like further assistance from the PHU to address these issues, please provide the name and contact details of your vaccination provider.	__________________________________
Is the online consent form for all four age-recommended vaccines useful?	◯ Yes<br>◯ No<br>◯ Don't know
How is it useful?	__________________________________
Why is it not useful?	__________________________________
Have you or will you be using the online consent form?	◯ Yes<br>◯ No<br>◯ Don't know
Can you tell us why you won't be using the online consent form?	__________________________________

Is the vaccination action plan useful?

◯ Yes
◯ No
◯ Don't know

How is it useful?

____

Why is it not useful?

____

Are the videos showing how to use the vaccination tracker, online consent form and vaccination action plan useful?

◯ Yes
◯ No
◯ Don't know

Why are they not useful?

____

Can you rank how useful the solutions are (1 = Most useful; 5 = Least useful)?

	1	2	3	4	5
Vaccination tracker	◯	◯	◯	◯	◯
Template online consent form	◯	◯	◯	◯	◯
Vaccination action plan	◯	◯	◯	◯	◯
Translated vaccine factsheets for residents	◯	◯	◯	◯	◯
Vaccine factsheets for residents and families	◯	◯	◯	◯	◯

What other resources or support would you have liked?

____

Which outcomes occurred for your facility as a result of this project? (please select all options that apply)

☐ Increased resident vaccination rates
☐ Increased awareness of vaccination by staff, residents and families
☐ Improved communication with residents and families (e.g. vaccination factsheets)
☐ Streamlined consent process
☐ Access to Australian Immunisation Register (AIR)
☐ Access to My Health Record (MHR)
☐ Increased knowledge of vaccination providers eg. nurse practitioners, staff who are authorised nurse immunisers (ANI)
☐ Improved access to vaccination providers
☐ Resident/family satisfaction with vaccination processes
☐ Upskilling of RACF staff (cold chain management, ANI training)
☐ Free authorised nurse immuniser (ANI) course through the Primary Health Network (PHN)
☐ Remind GPs or other vaccination providers to upload administered vaccines to AIR

How satisfied are you with the entire process (assessment, feeding back results, communication by the Public Health Unit)?	◯ Very satisfied<br>◯ Somewhat satisfied<br>◯ Neutral<br>◯ Somewhat dissatisfied<br>◯ Very dissatisfied
Can you explain why you are not satisfied with the process?	__________________________________
Do you have any suggestions on how to improve future projects?	__________________________________
